# Real-time and non-destructive hydrocarbon gas sensing using mid-infrared integrated photonic circuits

**DOI:** 10.1039/c9ra10058j

**Published:** 2020-02-19

**Authors:** Tiening Jin, Junchao Zhou, Pao Tai Lin

**Affiliations:** a Department of Electrical and Computer Engineering, Texas A&M University, College Station, Texas 77843, USA; b Department of Materials Science and Engineering, Texas A&M University, College Station, Texas 77843, USA; c Center for Remote Health Technologies and Systems, Texas A&M University, College Station, Texas 77843, USA

## Abstract

A chip-scale mid-infrared (mid-IR) sensor was developed for hydrocarbon gas detection. The sensor consisted of amorphous Si (a-Si) optical ridge waveguides that were fabricated by complementary metal–oxide–semiconductor (CMOS) processes. The waveguide exhibited a sharp fundamental mode through *λ* = 2.70 to 3.50 μm. Its sensing performance was characterized by measuring methane and acetylene. From the spectral mode attenuation, the characteristic C–H absorption bands associated with methane and acetylene were found at *λ* = 3.29–3.33 μm and *λ* = 3.00–3.06 μm, respectively. In addition, real-time methane and acetylene concentration monitoring was demonstrated at *λ* = 3.02 and 3.32 μm. Hence, the mid-IR waveguide sensor enabled an accurate and instantaneous analysis of hydrocarbon gas mixtures.

## 1. Introduction

Hydrocarbons are molecules consisting of carbon, hydrogen, and oxygen atoms. Hydrocarbons have different chemical and physical properties depending on their molecular structures and chemical bonds.^1,2^ Various hydrocarbon gases, such as methane (CH_4_), ethane (C_2_H_6_), ethylene (C_2_H_4_), and acetylene (C_2_H_2_), are the major components of natural gas. In addition to petroleum, hydrocarbon gases are extensively used in chemical syntheses and semiconductor processes. On the other hand, they react to form ground-level ozone in the atmosphere and become the major component of smog, which result in the greenhouse effect. In addition, the inhalation of hydrocarbon gases may pose several health risks, with short-term exposure potentially causing dizziness, intoxication, irritations, and anesthesia. Thus, the monitoring of hydrocarbon gases is not only essential in industry but is also critical in the prevention of pollution.

Various sensing methods have been explored to detect hydrocarbon gases, including catalysis, electro-chemistry, ultrasonics, semiconductors, and infrared absorption.^3–7^ In the oil industry, catalytic-type combustible sensors have been used to detect flammable hydrocarbon gas leaks. However, they have several drawbacks, such as sensor degradation and malfunction due to the harsh environments and frequently require oxygen to maintain detection accuracy.^8,9^ Solid-state sensors based on metal-oxide semiconductors (MOS) have shown high sensitivity and fast response times since their conductivity changes in the presence of an analyte gas. However, MOS sensors have poor selectivity and suffer from interference caused by background gases, such as ozone, water, and volatile organic compounds (VOCs).^10–12^ Another commonly used method, gas chromatograph (GC), is based on identifying the flow rate of analyte gas. However, GC requires an operation temperature that can exceed 150 °C to prevent the condensation of the gas compounds inside the device, which cause instability during the gas analysis because of thermal crosstalk. Moreover, the GC instrument are bulky and expend excessive amounts of energy, rendering them impractical for portable, on-site, and continuous gas detection.^13–19^ Therefore, it is critical to discover new sensing platforms that can detect hydrocarbon gases in a chip scale, with high specificity and sensitivity, and be compatible with CMOS for large volume manufacturing. A promising approach to achieve *in situ* and multiple hydrocarbon gas detection is mid-IR spectroscopy. Mid-IR is a spectral regime between wavelengths *λ* = 2.5 μm and 20 μm that overlap with the characteristic absorptions of various molecules, including CO_2_, CH_4_, NO, HCN, SO_2_, and VOCs. As such, mid-IR measurements provide both detection specificity and sensitivity for accurate and in-parallel gas identification. Hence, gas tracing through the mid-IR detection technique has been widely applied in the oil industry, as well as in environmental monitoring.^20–28^ Presently, however, acquiring infrared absorption spectra requires Fourier transform infrared spectroscopy (FTIR) or diffraction grating monochromators, both of which are benchtop instruments and thus are not suitable for portable or remote gas measurements. Alternatively, recent work in mid-IR planar photonics has shown that real-time detection on-a-chip is possible.^28,29^ Devices like pedestal waveguides, microring resonators, and opto-nanofluidics were able to recognize various chemicals by analyzing the spectral attenuation of the waveguide modes.

In this work, we propose integrated Si photonic circuits for the detection of important hydrocarbon gases, CH_4_ and C_2_H_2_. CH_4_ is the most common hydrocarbon gas used for domestic and commercial heating, and it is additionally used as an efficient cooking fuel worldwide. C_2_H_2_ is the simplest alkyne, and its worldwide production (over 150 million tons each year) exceeds that of any other organic compound.^30,31^ The photonic circuits were developed based on a-Si ridge waveguides, which were fabricated through the conventional CMOS process. Comparing to a rib waveguides, a ridge waveguide has a stronger evanescent field that increases the interaction between the probe mid-IR light and the molecules surrounding the waveguide surface, thus providing a higher sensitivity. The waveguide structures and mode profiles were designed and calculated by the two-dimensional finite difference method (FDM). Strong evanescent fields were observed on the top surface of the waveguide, which are critical for hydrocarbon gas detection. From the optical characterization, a bright and sharp waveguide mode was observed and a low optical loss of 1.74 dB cm^−1^ was obtained. To evaluate the sensing performance of the waveguide device, a test platform was built with a tunable mid-IR light, multiple gas injection system, and mid-IR signal detector. Gas detection was accomplished by correlating the waveguide spectral attenuation with the characteristic gas absorption bands. The fabricated mid-IR waveguide sensor was able to perform real-time analysis and monitoring of hydrocarbon gas mixtures.

## 2. Experimental

### 2.1 Device fabrication

**Fig. 1:**
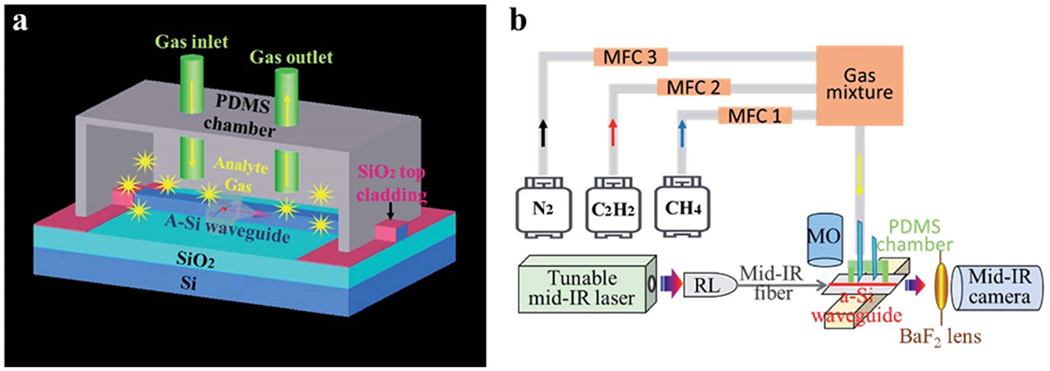
(a) The schematic of the waveguide sensor that consists of an a-Si waveguide and a PDMS micro gas chamber. The hydrocarbon gas mixture was injected into the chamber and then ejected using the inlet and outlet tubes. The waveguide evanescent field was absorbed by the gas molecules approaching to the waveguide surface. (b) The diagram of the gas sensing system. Mid-IR laser light was focused in a single mode fiber using a reflective lens (RL) and butt-coupled into the waveguide. The waveguide mode image and intensity were recorded by a camera placed after a BaF_2_ lens. The MFCs controlled the flow rate of N_2_, C_2_H_2_, and CH_4_ respectively. These gases were combined into a gas tube and then injected into the PDMS chamber.

As shown in Fig. 1a, the device mainly consists of two parts: an a-Si ridge waveguide and a (polydimethylsiloxane) PDMS micro-gas chamber. When the hydrocarbon gases were injected into the PDMS chamber, the mid-IR light going through the waveguide was absorbed by the analytes according to their C–H bond vibration mode. In the fabrication process, first, a 1 μm thick a-Si thin film was deposited on the Si wafer that had a 3 μm thick SiO_2_ layer prepared by plasma-enhanced chemical vapor deposition (PECVD). The precursor gas for the a-Si deposition was SiH_4_, and the deposition temperature was 200 °C. Then, a 50 nm thick layer of Cr mask was patterned onto the a-Si layer by lithography, electron beam evaporation, and lift-off process, sequentially. The waveguide structure was transferred to the a-Si layer by reactive ion etching (RIE). SF_6_ was used for selective Si etching. Finally, the Cr mask and the organic residue on the device surface were removed by rinsing the surface with a ceric ammonium nitrate solution followed by oxygen plasma ashing. After waveguide fabrication, a 2 μm thick SiO_2_ layer was deposited on both ends of the waveguides by PECVD to prevent the PDMS chamber from contacting the waveguide surface. PDMS has absorption bands in the mid-IR range due to its O–H, C–H, and other chemical bonds. The waveguide light would be absorbed if the PDMS chamber was directly attached to the waveguide surface.

### 2.2 Optical characterization system

#### Sensing system set-up.

To characterize the optical performance of the waveguides, a mid-IR test station was assembled and is shown in Fig. 1b. The light source was a pulsed laser with a 150 kHz pulse repetition rate, a 10 ns pulse duration, and a 150 mW average power. Using a reflective lens, the probe light was collimated into a fluoride fiber with a 9 μm core and 125 μm cladding and then butt-coupled into the waveguide. The fine alignment between the optical fiber and the a-Si waveguide was monitored by a microscope equipped with a long working distance, 10× objective lens. The gas delivery subsystem had three mass flow controllers (MFC) to regulate the flow rates of C_2_H_2_, CH_4_, and N_2_. The analyte concentration was adjusting by tuning the ratio of the three gas flow rates. The gas sample was delivered to the sealed PDMS chamber placed on top of the waveguide sensor, in which the a-Si waveguides were exposed to the gas analytes. The mid-IR signals from the waveguides were focused by a calcium fluoride biconvex lens with a 25 mm focal length, and imaged by a liquid nitrogen-cooled InSb camera.

## 3. Results and discussion

### 3.1 Morphology of the device

**Fig. 2:**
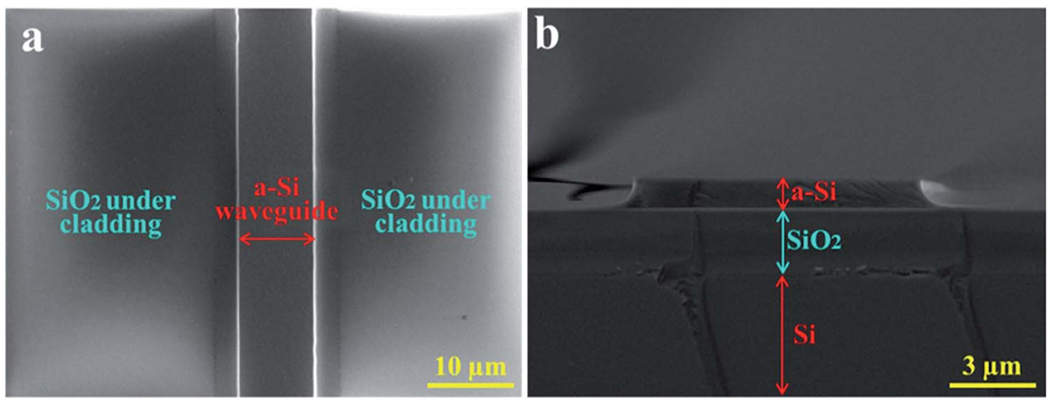
The SEM (a) top and (b) cross-sectional images of the a-Si waveguide. It is 10 μm wide and 1 μm high on a 3 μm thick SiO_2_ under-cladding layer. The substrate is Si wafer.

The morphology of the fabricated a-Si waveguides was first inspected by scanning electron microscopy (SEM). Fig. 2a shows the top surface of the a-Si waveguide sitting on top of the SiO_2_ under-cladding. The structure was clearly defined, without defects or particles found along the edges. The top surface was smooth, indicating that no depletion damage was introduced during the RIE etching process. From the cross-sectional SEM image in Fig. 2b, the waveguide structure was 10 μm wide and 1 μm high and it had a well-cleaved end facet, which was critical to minimize the optical loss caused by scattering. The 10 μm waveguide width improved the butt-coupling efficiency between the 9 μm core diameter mid-IR fiber and the waveguide.

### 3.2 Optical simulation and characterization

#### Optical property simulation.

**Fig. 3:**
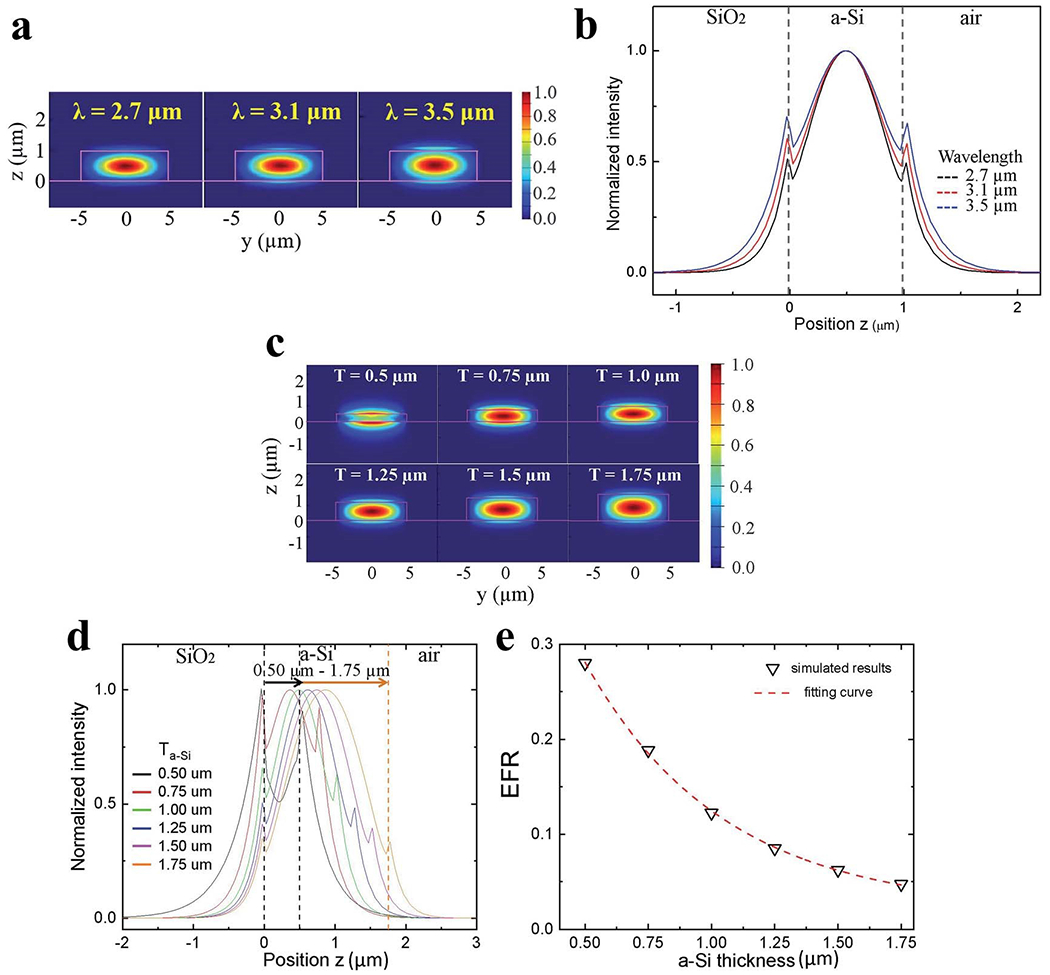
(a) The optical field of the mid-IR waveguide calculated between *λ* = 2.7–3.5 μm. Fundamental modes with similar ellipsoid intensity distributions were resolved in the a-Si layer. (b) The calculated 1-D intensity profiles along the *z*-axis where the evanescent field expanded beyond and below the a-Si area. (c) The calculated optical field at *λ* = 3.30 μm when the *T*_a-si_ increased from 0.5 to 1.75 μm. (d) The corresponding ID intensity profiles along the *z* direction at *y* = 0 μm. (e) The calculated EFR *versus T*_a-Si_ at *λ* = 3.30 μm.

The waveguide modes were numerically calculated over the spectrum by the two-dimensional FDM. In the simulation, the device structure was based on the SEM inspection results, where the a-Si waveguide was 10 μm wide and 1 μm high. The refractive indexes for a-Si and SiO_2_ are 3.44 and 1.45, respectively. A 12 μm × 8 μm light source was chosen to excite the waveguide mode since its size is comparable to the mid-IR fiber of a 9 μm core. Fig. 3a illustrates the optical field distribution of the waveguide mode calculated between *λ* = 2.70 and 3.50 μm. A fundamental mode with a similar ellipsoid intensity profile was obtained in the a-Si waveguide center. The evanescent field existed on the top (*z* ≥ 0 μm) and bottom (*z* ≤ −1 μm) surfaces of the a-Si layer, which was associated with the field distribution of the transverse magnetic (TM) polarization mode. The evanescent field gradually increased as the probe light shifted to longer wavelengths. To better analyze the mode properties, the calculated 1-D intensity profiles along the *z*-axis were plotted in Fig. 3c. A Gaussian intensity profile corresponding to a fundamental mode was obtained due to the relatively high refractive index contrast between the a-Si waveguide core and the SiO_2_ cladding. For longer wavelengths, the light field distribution tended to expand out of the a-Si layer (*z* ≥ 0 μm). Such a structure is suitable for mid-IR waveguide sensing applications because the gas molecules approaching the waveguide surface absorb the evanescent field according to their characteristic absorption bands. Thus, the composition and concentration of the gas mixtures can be identified by analyzing the spectral attenuation of the waveguide mode. In addition, the preservation of the fundamental mode over a wide spectral range between 2.70–3.50 μm improved the sensing. Excitation of higher order modes would change the mode profile and the evanescent field distribution, which may consequently lead to false sensing signals.

For the evanescent field sensing, the thickness of the a-Si ridge waveguide, *T*_a-si_, is critical because *T*_a-si_ determines the strength of the evanescent field and the sensitivity. Sensing performance at various *T*_a-si_ were simulated and analyzed. Fig. 3c showed the waveguide TM modes at *λ* = 3.30 μm when the *T*_a-si_ increased from 0.5 μm to 1.75 μm. At *T*_a-si_ = 0.5 μm, the a-Si layer was too thin to effectively confine the mid-IR and strong fields were found in the interface between the waveguide and the cladding layers. As the *T*_a-si_ gradually increased to 1.75 μm, the confinement of the mid-IR became stronger and the evanescent wave became weaker. To better visualize the optical field profiles at different *T*_a-si_, 1-D intensity distributions along the *z*-axis {*y* = 0) are plotted in Fig. 3d. As *T*_a-si_ increased, the evanescent field decreased and the waveguide mode shifted toward the center of the a-Si layer. Fig. 3e is the plot of the calculated evanescent field ratio (EFR) at different *T*_a-si_, where a better sensitivity was obtained at a high EFR.^32–34^ As the *T*_a-si_ decreased from 1.75 μm to 0.5 μm, the EFR increased sharply from 0.05 to 0.28 indicating a significant improvement of the sensitivity. Nerveless, the coupling efficiency between the fiber and waveguide dropped as the *T*_a-si_ decreased. Therefore, an optimized of *T*_a-si_ = 1 μm was selected in this study.

#### Optical characterization.

**Fig. 4:**
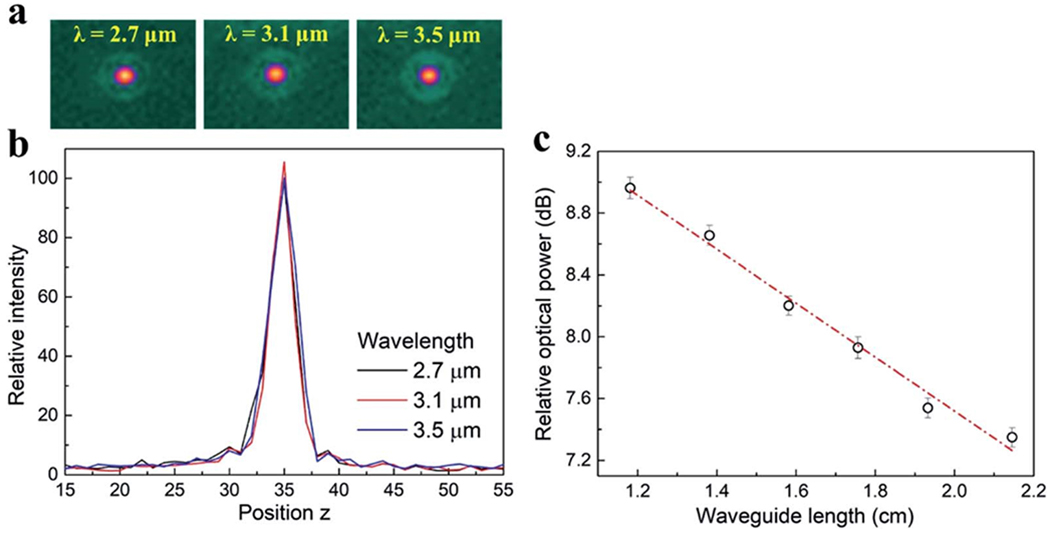
(a) The waveguide mode images captured between *λ* = 27–3.5 μm. A fundamental mode was clearly observed over a broad spectral range. (b) The 1-D intensity profile of the waveguide mode extrapolated along the *y*-direction. This resolved Gaussian like profile represented a fundamental mode. (c) The relative optical powers measured from waveguides with different lengths. The optical loss was determined to be 1.74 dB cm^−1^ by fitting the mode intensity attenuation at *λ* = 3.1 μm.

The optical performance of the a-Si waveguide was characterized by the mid-IR testing station. The mode images and mode intensity attenuations measured at different waveguide lengths are shown in Fig. 4. In Fig. 4a, a fundamental mode is clearly observed over the spectral range from *λ* = 2.70 to 3.50 μm. The mode profiles remained the same at different wavelengths. No scattering was found in the captured mode images, indicating that the waveguide had a smooth surface and a sharp interface between the a-Si and the SiO_2_ layers. The higher refractive index of a-Si compared to SiO_2_ contributes to an efficient lightwave guiding. The 1-D mode intensity profiles were then extrapolated and are illustrated in Fig. 4b. A well-resolved Gaussian profile corresponding to a fundamental mode was found over λ = 2.70 to 3.50 μm, which is consistent with the simulated mode profiles displayed in Fig. 3b. The mode intensity of waveguides with different lengths was measured to characterize the optical loss. By fitting the mode intensity attenuation shown in Fig. 4c, a loss of 1.74 dB cm^−1^ was obtained at *λ* = 3.10 μm. The resolved low optical loss is attributed to the intrinsic mid-IR transparency of the deposited a-Si. The fabrication process created smooth device surfaces which also minimized the optical loss. In addition, the scattering loss caused by surface roughness was lower at longer wavelengths, such as mid-IR, since Rayleigh scattering is proportional to 1/*λ*^4^. Thus, the a-Si waveguides are an ideal platform for chip-scale mid-IR sensing.

### 3.3 Sensing tests

**Fig. 5:**
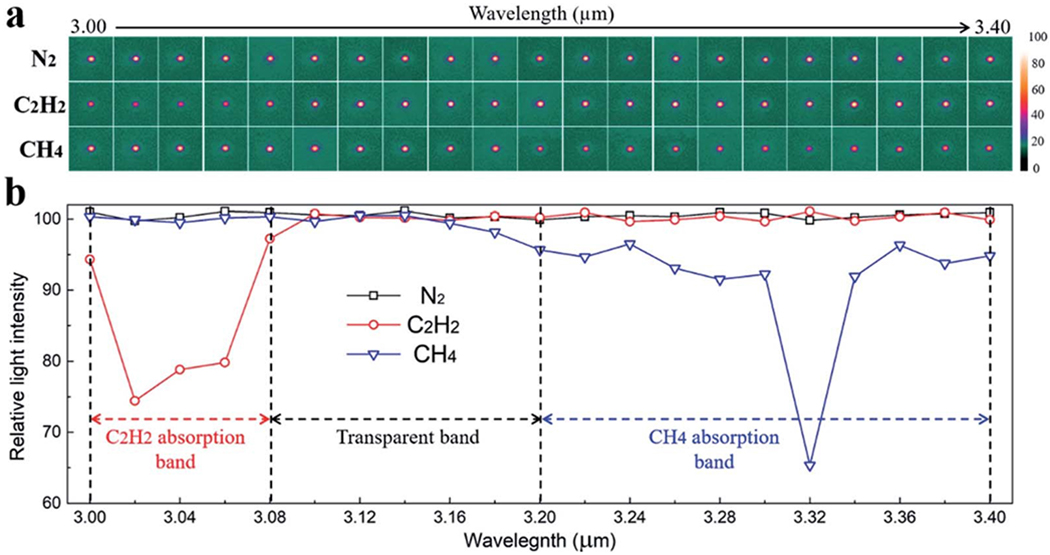
(a) The images of the captured waveguide modes when N_2_, C_2_H_2_, and CH_4_ gases were injected into the PDMS chamber. The wavelength was scanned between *λ* = 3.0–3.4 μm. (b) The waveguide mode intensities when N_2_, C_2_H_2_, and CH_4_ were applied. Strong intensity attenuation was found between *λ* = 3.00 to 3.08 μm for C_2_H_2_ and *λ* = 3.29 to 3.33 μm for CH_4_.

Two hydrocarbon gases, C_2_H_2_ and CH_4_, were selected to evaluate the sensing performance of the a-Si waveguide. The wavelength of the probe light was sequentially scanned from *λ* = 3.00 to 3.40 μm, a spectral region that included the characteristic C-H absorptions caused by C_2_H_2_ and CH_4_. Fig. 5a shows the captured waveguide mode images upon the injection of different gases into the PDMS chamber. When N_2_ was injected, the mode intensity was strong through the scanning range because the N≡N bond is IR inactive. When C_2_H_2_ was delivered into the chamber, the light mode notably faded between *λ* = 3.00 and 3.08 μm. After *λ* = 3.08 μm, the light mode became bright again. Once CH_4_ was presented, the light mode intensity remained strong over the spectrum except near *λ* = 3.32 μm. The results revealed that C_2_H_2_ and CH_4_ have distinguishable absorption properties within this scanning region. To better analyze these two gas absorption results, the waveguide mode intensity at discrete wavelengths was recorded and is depicted in Fig. 5b. For C_2_H_2_, significant light attenuation was found from *λ* = 3.00 to 3.08 μm, which was caused by its asymmetric C–H stretching.^35,36^ On the other hand, for CH_4_, the mode intensity dropped between *λ* = 3.18 and 3.40 μm due to the asymmetric C–H vibration, *ν*_3_. The absorptions found at *λ* = 3.32–3.40 μm and 3.18–3.30 μm were assigned to the prominent R branch and the mild P branch of CH_4_, respectively.^37,38^ Hence, the mid-IR waveguide sensor can accurately differentiate C_2_H_2_ and CH_4_ through their dissimilar C–H absorption spectra.

**Fig. 6:**
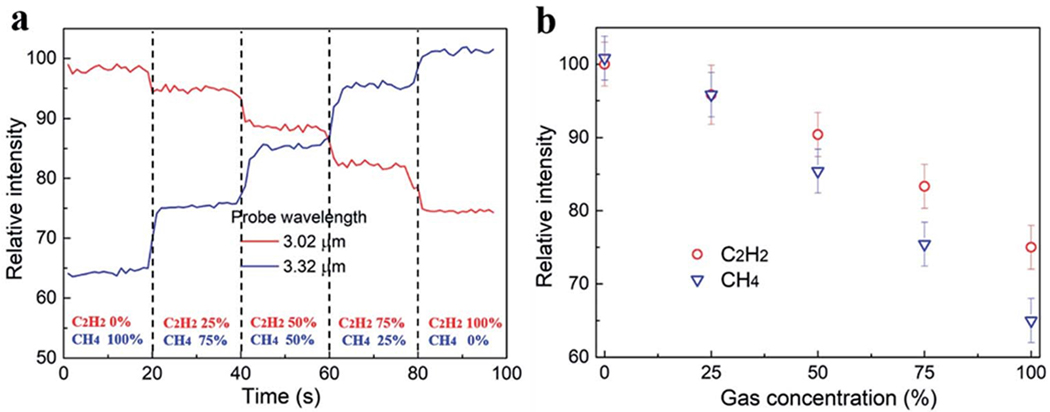
(a) The real-time monitoring of C_2_H_2_/CH_4_ mixture using the a-Si waveguide. The concentration of CH_4_ was decreased from 100% to 0%, and the C_2_H_2_ was increased from 0% to 100% by a step of 25%. The wavelengths were *λ* = 3.02 and 3.32 μm. The waveguide mode intensities changed sharply as the C_2_H_2_ and CH_4_ concentrations varied. (b) The mode intensities measured at various C_2_H_2_/CH_4_ concentrations. A monotonic dependence between the waveguide mode intensity and C_2_H_2_/CH_4_ concentrations was found.

The real-time waveguide sensing test was carried out by injecting C_2_H_2_ and CH_4_ mixtures into the PDMS chamber. The sequence of the CH_4_/C_2_H_2_ concentrations were 0%, 25%, 50%, 75%, and 100%. Here, the concentration ratios were defined by the two gas flow rates controlled by the MFCs. In parallel, the laser wavelength was tuned to 3.02 μm and 3.32 μm, respectively. C_2_H_2_ attenuated the light intensely at *λ* = 3.02 μm, while CH_4_ was transparent at that wavelength. In contrast, the light at *λ* = 3.32 μm was only absorbed by CH_4_, and not by C_2_H_2_. As shown in Fig. 6a, abrupt mode intensity changes were observed between different CH_4_/C_2_H_2_ mixtures. In addition, both wavelengths were able to monitor the variation of the concentrations instantaneously. The light intensity at *λ* = 3.32 μm decreased as the CH_4_ concentration increased. Equivalently, the intensity at *λ* = 3.02 μm increased when the C_2_H_2_ concentration decreased. Fig. 6b plots the waveguide mode intensities measured at various CH_4_/C_2_H_2_ concentrations where a monotonic dependence was observed. The device sensitivity *S* can be expressed by eqn (1)^32–34^

(1) $$S=-\eta \varepsilon lI_{0}e^{\left(-\eta \varepsilon Cl-\alpha l\right)}$$

where *I*_0_ is the input power of 20 mW, *ε* is the absorption coefficient, which is 12.8 atm^−1^ cm^−1^ at *λ* = 3.32 μm for CH_4_ and 10.2 atm^−1^ cm^−1^ at *λ* = 3.02 μm for C_2_H_2_, *C* and concentration of the hydrocarbon gases, *α* is the intrinsic optical loss of the waveguide device, *l* is the length of the waveguide of 1 cm, and *η* is the EFR. The mid-IR intensity attenuation was attributed by the intrinsic waveguide optical loss of 1.74 dB cm^−1^ and the gas absorption losses. Applying eqn (1), *S* is −5.4 mW atm^−1^ for 5% CH_4_ at *λ* = 3.32 μm. For 5% C_2_H_2_, *S* is −4.4 mW atm^−1^ at *λ* = 3.02. The sensitivity can be further improved by decreasing the waveguide height and increasing the waveguide length. The results confirmed that the a-Si waveguide sensor was capable of performing *in situ* and quantitative hydrocarbon detection.

## 4. Conclusions

A mid-IR a-Si waveguide sensor was demonstrated for non-destructive and real-time hydrocarbon gas detection. The waveguides were fabricated by CMOS processes. A clear waveguide fundamental mode was observed through *λ* = 2.70 to 3.50 μm, and a low optical loss of 1.74 dB cm^−1^ was obtained. Two hydrocarbon gases, C_2_H_2_ and CH_4_, were applied to test the waveguide sensing performance. A strong mode intensity attenuation was found between *λ* = 3.00–3.06 μm when C_2_H_2_ was applied, and between λ = *λ* = 3.29 to 3.33 μm when CH_4_ was used. The spectral mode variation corresponded to the different characteristic absorption bands caused by C–H stretching vibrations. Therefore, the mid-IR waveguide device provided a chip-scale sensor for *in situ* and remote hydrocarbon detection.
